# Hot-Air-Assisted Radiofrequency Drying of Olive Pomace and Its Effect on the Quality of Olive Pomace Oil

**DOI:** 10.3390/foods12183515

**Published:** 2023-09-21

**Authors:** Sinem Turan, Aysel Elik Demir, Fahrettin Göğüş, Derya Koçak Yanık

**Affiliations:** 1 Department of Food Engineering, Engineering Faculty, University of Gaziantep, Gaziantep 27310, Türkiye; sinemturaan@gmail.com (S.T.); fahret@gantep.edu.tr (F.G.); 2 Department of Food Technology, Vocational School of Technical Sciences at Mersin Tarsus Organized Industrial Zone, Tarsus University, Mersin 33100, Türkiye; ayselelik@tarsus.edu.tr; 3 Department of Food Engineering, Faculty of Agriculture, Eskişehir Osmangazi University, Eskişehir 26160, Türkiye

**Keywords:** olive pomace, radiofrequency drying, olive pomace oil, mathematical modeling

## Abstract

In this study, the drying of olive pomace in a hot-air-assisted radio frequency system (HA–RF) was conducted, and its effects on crude olive pomace oil quality were investigated. In this respect, the effects of radiofrequency electrode distance (90, 105 and 120 mm), sample thickness (2.5, 5, 7.5 and 10 cm) and compaction density (~0.45, ~0.60 and ~0.82 g/cm^3^) on drying rate have been evaluated. The best drying, with a higher drying efficiency, was obtained with 1 kg of sample weight and a 10 cm product thickness, ~0.45 g/cm^3^ compaction density and 105 mm electrode distance. Moreover, the results showed that the compaction density significantly affects the drying rate. The drying time was prolonged by approximately four times by increasing the compaction density from ~0.45 to ~0.82 g/cm^3^. The drying rate of olive pomace in HA–RF drying was compared with drying performed using hot air (HA) and radiofrequency (RF). The results revealed that HA–RF application reduced the drying time by almost 1.7 times compared to hot air drying and by about 2.7 times compared to radiofrequency. The peroxide value, free fatty acid content, p-anisidine value, polyaromatic hydrocarbon content, L*, a*, b*, chlorophyll and total carotenoid content of the oil extracted from the olive pomace dried under the best drying conditions were 1.09%, 12.2 meq O_2_/kg oil, 3.01, <1 ppb, 38.6, 7.5, 62.56, 105.25 mg pheophytin a/kg oil, 2.85 mg/kg oil, respectively. The drying of olive pomace in a hot-air-assisted radio frequency system could be an alternative way to ensure the safe and rapid drying of olive pomace.

## 1. Introduction

Olive pomace is a (semi) solid food industry waste generated during the olive oil extraction process. Morocco, Spain, Greece, Italy, Tunisia, Syria, Turkey and some other Mediterranean countries are the main producers of olive oil and olive pomace [1]. More than half of the crude olive pomace is water and the rest contains olive seed, pulp, skin and some oil. The quantity, chemical properties and moisture content of olive pomace highly depend on the extraction method used during olive oil extraction and the fruit cultivar. Even if olive oil extraction mainly adapts to a two-phase decanter system in European countries, a three-phase decanter is still widely used in some countries. While the moisture of the pomace is changing between 40 and 50% wet basis (wb) in a three-phase extraction system, it changes between 60 and 70% wb for the two-phase extraction system [2]. Hence, the drying of olive pomace is required for its additional usage. 

As indicated, the current use of oily olive pomace is mainly for the extraction of residual oil while its use as a fertilizer or fuel is an additional benefit [3]. The market value of this waste product is determined with respect to its oil content. After the crude olive pomace oil is refined, it can be mixed with the appropriate amount of extra virgin olive oil and offered for human consumption. Therefore, the quality of crude olive pomace oil is the most important criterium determining the cost of the refining process. Due to the high moisture content of olive pomace, it is essential to dry the pomace before oil extraction. Therefore, the quality of the crude olive pomace oil considerably depends on the drying stage that has to take place prior to the solvent extraction of the oil [4,5]. Since fossil fuel, or dry oil-free olive pomace, is traditionally used as a fuel for the drying of wet olive pomace in the industry, high emissions of greenhouse gases are caused. The general practice used in olive pomace drying is based on the direct exposure of the olive pomace to a hot gas stream in a rotary dryer, in which temperatures can reach up to 400–800 °C [6]. The crude oil extracted from the pomace dried using this conventional method is dark in color, high in acidity and oxidized compounds and has a characteristic off-odor. Moreover, this conventional drying technique causes an increase in polycyclic aromatic hydrocarbons (PAHs) because of the direct contact of combustion fumes with the pomace [5,6,7]. These are some important obstacles to obtaining edible olive pomace oil of a reasonable cost, safety and quality from olive pomace dried via conventional drying. The problems and disadvantages of conventional drying can be solved with the application of novel and effective drying processes that might have the potential to be integrated on an industrial scale. In addition, the use of alternative methods, such as RF with lower CO_2_ emissions, for the drying of the olive pomace helps to reduce environmental pollution.

There have been many studies in the literature on drying kinetics in recent years [8,9,10]. Even though several olive pomace drying studies are available in the literature, the majority of them have focused on drying kinetics [11,12,13]. There are only a few studies that associate drying systems with the final oil quality [7,14,15]. 

Radio frequency (RF) heating is one of the dielectric heating methods that provides the volumetric heating of materials. RF-assisted heating has recently been proposed as a promising drying method to obtain dried fruits and vegetables and their powders due to its high efficiency, quick processing and longer penetration depth. The RF drying of carrots [16], black carrot pomace [17], avocado [18], carrot [19] and potato [20] are some of these recent applications. Although intensive studies have been carried out on RF drying systems in recent years, there is a lack of information on its effects on the drying characteristics of samples containing oil, and oil quality. Additionally, the RF drying technique can provide high-speed, efficient, high-quality and convenient drying via combination with hot air (hot-air-assisted RF) [21], and this can be easily adapted to a continuous industrial scale process. The biggest potential challenge of the RF drying system is its high initial investment cost [22]. However, shortening the drying time is thought to compensate for this disadvantage.

This study aims to figure out the effect of parameters such as electrode distance, sample thickness and compaction density on the drying rate of olive pomace in hot-air-assisted radiofrequency (HA–RF) drying. The drying behavior of the olive pomace under HA–RF has been compared with that dried using hot air and radiofrequency. Moreover, this study aims to discuss the effect of HA–RF drying on the quality characteristics of the oil obtained from dried pomace. 

## 2. Materials and Methods

### 2.1. Materials

This study was carried out on the olive variety Nizip yağlık. Olive pomace (56.0% moisture (wet basis, wb), 6.32% oil in dry matter) from a three-phase decanter system was obtained from a local producer in Gaziantep, Turkey. In total, 50 kg of the olive pomace was stored at 5 °C in polyethylene bags until the drying experiments were performed. All HA–RF drying experiments were performed using this olive pomace. Standard chromatographic grade benzo[a]pyrene and fatty acid methyl ester (FAME Supelco^®^ 37 mixture) were purchased from SigmaAldrich (St. Louis, MO, USA). Solvents (n-hexane and n-heptane) were used for GC analysis. Other reagents and solvents were of analytical grade. Ultra-pure water for HPLC analysis was purified using a Millipore system (Milli-Q system, Millipore, Bedford, MA, USA).

### 2.2. Hot-Air-Assisted Radiofrequency Drying

A hot-air-assisted radiofrequency drying system (Sonar, Izmir, Turkey) described by Elik [17] was used in the drying experiments. For drying tests, the HA–RF drying system was turned on and made ready for drying one hour before the start. In all drying experiments, the air temperature and flow rate were kept constant at 50 °C and 1.5 m/s, respectively. The weight was recorded (electronic balance, SW, CAS, Korea) by taking out the tray from the system every 10 min. The drying process was carried out until the moisture content of olive pomace was reduced to 0.04 ± 0.01 g water/g dry solids (ds). Thus, the final moisture content of the olive pomace reduced to less than 5% (wb) as in industrial processes.

Various electrode distances (90–120 mm) were studied to determine their effect on drying characteristics. Olive pomace (1000 g) was placed in a polypropylene tray (28.5 cm × 19.5 cm × 10 cm) with perforated side and bottom walls, which allow air passage through the product, and then was subjected to HA–RF drying. The effect of sample thickness on drying behavior was studied in the range from 2.5 to 10 cm with 1000 g of olive pomace at 105 mm electrode distance (determined in previous step). In order to determine the effect of RF application, three more separate drying experiments with 1000 g of olive pomace at 2.5 cm sample thickness and 105 mm electrode distance were performed with HA–RF, RF and HA. Additionally, the effect of compaction density (porosity) on the HA–RF drying of olive pomace was investigated. Firstly, wet olive pomace (2500 g) was loosely filled (without compaction) into a rectangular container (L: 28.5 cm and W: 19.5 cm) with a thickness of 10 cm (volume of 5557.5 cm^3^, the compaction density of ~0.45 g/cm^3^) in bulk. The compaction density (g/cm^3^) of this bulk olive pomace was calculated by dividing the mass of the sample (g) by its volume (cm^3^). Then, in order to obtain a higher compaction density, the same amount of wet olive pomace (2500 g) was packed into 7.5 cm (~0.60 g/cm^3^) and 5.5 cm (~0.82 g/cm^3^) thicknesses by compression from the top surface of the sample.

### 2.3. Drying Behavior

Drying curves were modeled with the drying data of moist olive pomace dried using HA–RF, RF and HA drying methods. The weight data recorded during drying studies were used in modeling drying curves.

The following equation was used in the calculation of the moisture ratio (*MR*) of the samples [23]:

(1) $$MR=\frac{M_{t}-M_{e}}{M_{0}-M_{e}}=\frac{8}{\pi^{2}}\exp(-\frac{\pi^{2}}{4}\frac{D_{eff}\;t}{L^{2}})$$

where *M_t_* is the moisture content (dry basis, db) at time *t*, *M_e_* is the equilibrium moisture content (db), *M*_0_ is the initial moisture content (db), *D_eff_* is the effective moisture diffusivity (m^2^/s), *L* is the thickness of the slab and *t* is the drying time (s).

Equation (1) is evaluated numerically for Fourier number (*F*_0_ = *Dt*/*L*^2^) for diffusion and can be rewritten as [24]:

(2) $$MR=\frac{8}{\pi^{2}}\exp(-\frac{\pi^{2}}{4}F_{0})$$

(3) $$F_{0}=-0.405\;In\left(MR\right)-0.0851$$

(4) $${\;D}_{eff}=\frac{F_{0}}{t/L^{2}}$$

*D_eff_* was estimated by substituting the positive values of *F*_0_ and the drying time along with half thickness in Equation (4) for the corresponding average moisture content.

The drying rate (*DR*, g water/(g ds·min)) was calculated using the equation given below [24]:

(5) $$DR=\frac{{M_{t1}-M}_{t2}}{t2-t1}$$

where *M_t_*_1_ and *M_t_*_2_ are the moisture content at time *t*1 and *t*2 in g water/g dry solid and *t* is the drying time (min).

### 2.4. Moisture Content and Oil Content Determination

The olive pomace (10 g) was kept in an oven at 130 °C until constant weight to determine the moisture content. The total oil content of the olive pomace was determined via the Soxhlet method using n-hexane as a solvent.

Extraction of olive pomace oil

Olive pomace oil was extracted using a cold extraction method with n-hexane from the olive pomace samples dried under the conditions (105 mm electrode distance, 10 cm of sample thickness) where the highest effective diffusivity was obtained. After that, the hexane was evaporated at 50 °C by a rotary vacuum evaporator (Heidolph, Schwabach, Germany) to obtain crude olive pomace oil for further quality analysis. The final moisture content of the olive pomace used for oil extraction was below 5%.

### 2.5. Oil Quality Evaluation

The peroxide value (PV), free fatty acid (FFA) content and p-anisidine value (p-AnV) of olive pomace oil were determined using methods described in AOCS Cd 8–53 [25], AOCS Ca 5a-40 [25] and AOCS Cd 18–90 [25], respectively. The total oxidation value (Totox) of the oil was calculated from the PV and p-AnV, as given in following equation [26].

Totox value = 2PV + pAnV(6)

All analyses were performed in triplicate. The results were presented as averages with their standard deviations.

Spectrophotometric examination in the ultraviolet (K_232_ and K_270_ values)

UV absorbances at 232 and 270 nm of oil were determined according to IOC [27]. Then, 0.25 g (to the nearest 1 mg) of oil was taken into a 25 mL graduated flask and the volume was made up to the mark with cyclohexane and homogenized. Then, the absorbance was measured using a spectrophotometer (Optima, SP-3000nano, Tokyo, Japan) at 232 and 270 nm. 

### 2.6. Color Measurement

The color of olive pomace oil was measured using a Hunter-Lab ColorFlex calorimeter. The measurements were performed at an observer angle of 10° and daylight D65. The L* value indicates the lightness, the a* value indicates greenness (−*a*) or redness (+*a*) and the b* value indicates the blueness (−*b*) or yellowness (+*b*) value. Hue angle (H*) and chroma (C*) values were calculated from L*, a* and b* values using the following equations.

(7) $$H^{*}={tan}^{-1}\left(b^{*}/a^{*}\right)$$

(8) $$C^{*}=\sqrt{\left((a^{*}{)}^{2}+(b^{*}{)}^{2}\right)}$$

### 2.7. Determination of Fatty Acid Composition

First, the fatty acids of pomace oil were converted into methyl esters as specified in IOC [28]. After methylation, 1 μL of FAME was automatically injected into a gas chromatograph. The Agilent 7890A GC (Agilent Technology, Santa Clara, CA, USA) is equipped with a split/splitless injector, a flame ionization detector and an HP-88 capillary column (88% Cianopropylaryl 100 m × 0.250 mm ID × 0.20 μm) and was used for GC analysis. The temperature program given by Yanık [6] was applied during the analysis. FAMEs were identified by comparing the retention time of the standard mixture with 37 FAME.

### 2.8. Determination of the Chlorophyll and Total Carotenoid Contents

The spectrophotometric method described in AOCS Cc 13k-13 [25] was used to determine the chlorophyll content. The absorbance of olive pomace oil was measured at 630, 670 and 710 nm against air. The content of chlorophyll pigments was calculated as pheophytin a in mg/kg of oil using the following Equation.

(9) $$C=kx\frac{A_{670}-0.5(A_{630}+A_{710})}{L}$$

where *k* is a constant factor, 34.53 [29] and *L* is the light path of the spectrophotometer cell (cm). 

The total carotenoid content of olive pomace oil was calculated as described by Gao et al. [30]. Briefly, a 0.5 g oil sample was dissolved in 4 mL of hexane. The absorbance of the solution was measured at 445 nm using n-hexane as a blank. The total carotenoid content was calculated using the following equation.

(10) $$Total\;Carotenoid\;(mg/kg)=\frac{A_{445}\times V\times 10^{6}}{2500\times m\times 100\times d}$$

where *d*: cell diameter (cm); *V*: volume of solution (mL); *m*: mass of oil sample; and 2500 dL g^−1^cm^−1^ is the specific extinction coefficient of carotenoids at 445 nm in n-hexane [31]. 

### 2.9. Determination of Benzo(a)pyrene

PAHs were extracted using the SPE method, according to the procedure defined by Moret and Conte [32]. After that, benzo(a)pyrene (BaP) content in the extract was determined using Shimadzu Prominence/LC-20 AB HPLC as described by Yanık [6].

## 3. Results and Discussion

### 3.1. Effect of Electrode Gap, Sample Thickness and Compaction Density on Drying Behavior of Olive Pomace 

**Figure 1:**
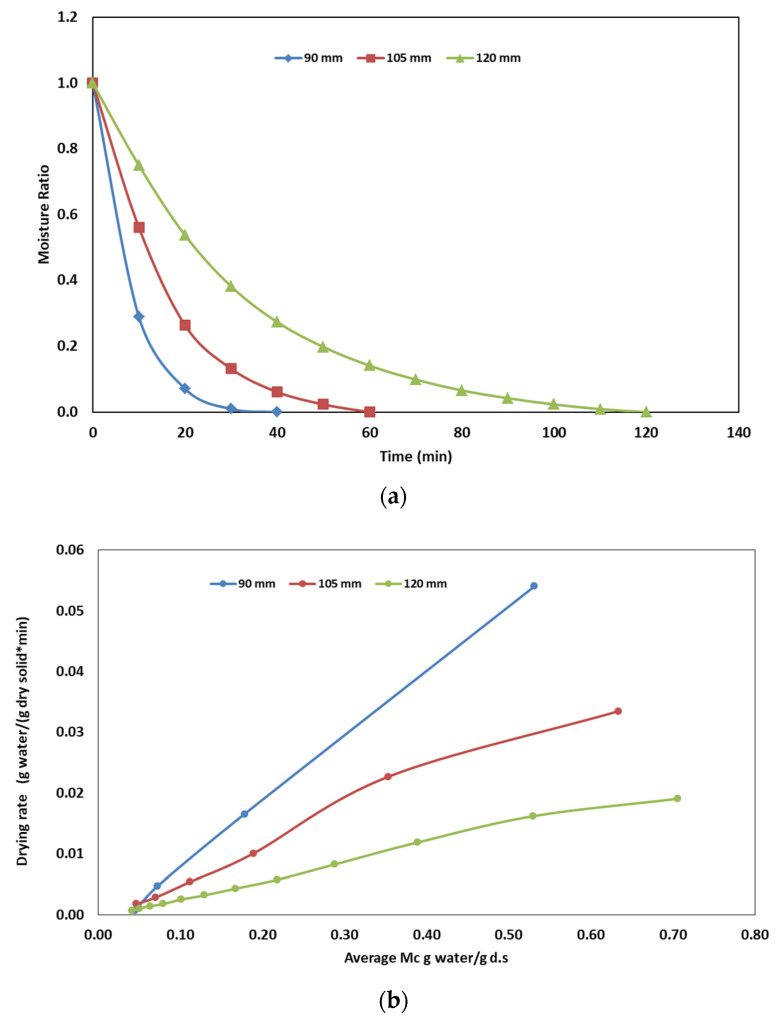
Effect of electrode distance on (**a**) drying behavior of olive pomace, (**b**) drying rate.

The RF electrode distance is one of the important factors affecting the drying behavior of samples under HA–RF drying. The effects of electrode distance (90, 105 and 120 mm) on the moisture curve and drying rate of olive pomace are given in Figure 1a,b, respectively. The sample weight and sample thickness were kept constant (1000 g and 7.5 cm samples) in these studies and just the electrode distance was changed. It was seen that the shorter the electrode distance, the higher the drying rate. According to Elik [17], a shorter electrode distance increases the drying rate because of the more intense electrical field. Also, Jiao et al. [33] reported that the drying rate increases with a shorter electrode gap during the HA–RF drying of peanuts. 

**Figure 2:**
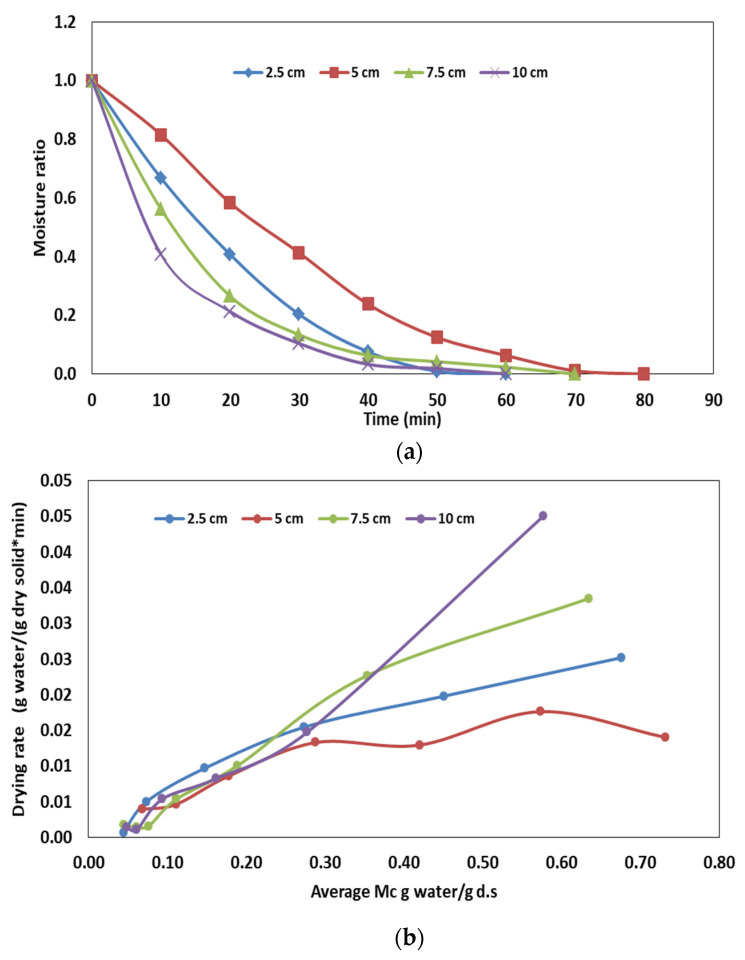
Effect of sample thickness on (**a**) drying behavior of olive pomace, (**b**) drying rate.

The effect of sample thickness (2–10 cm) on the drying curve and drying rate of olive pomace following HA–RF drying is given in Figure 2a,b, respectively. At sample thicknesses of 2.5, 5, 7.5 and 10 cm, the drying times to achieve the target moisture content (0.04 ± 0.01 g water/g ds) were 60, 80, 70 and 60 min, respectively. The results showed that the increase in the sample thickness from 5 cm to 10 cm caused an increase in the drying rate, providing a shorter drying time. In RF-drying systems, an increase in sample thickness generally results in a decrease in drying time due to improvements in the drying rate. This behavior can be explained by the greater wave adsorption due to the increase in sample thickness and decrease in the gap between the sample surface and upper electrode in the HA–RF drying system [34]. On the other hand, as it was found in this study, the drying time was the same for both sample thicknesses of 2.5 and 10 cm. In thin samples, while RF heating provided volumetric heating, HA removed the surface water much more efficiently due to the decrease in the moisture diffusion path from the interior to the surface [35]. Therefore, despite absorbing less electrical energy in a sample thickness of 2.5 cm, HA may have had more effect together with RF on the drying rate and so the drying time decreased. With the sample thickness of 2.5 cm, however, RF became the dominant mechanism with increasing thickness in determining the drying rate. Similarly, a previous study conducted by Hou et al. [36] on RF drying reported that the drying rate increased in the thicker sample. 

**Figure 3:**
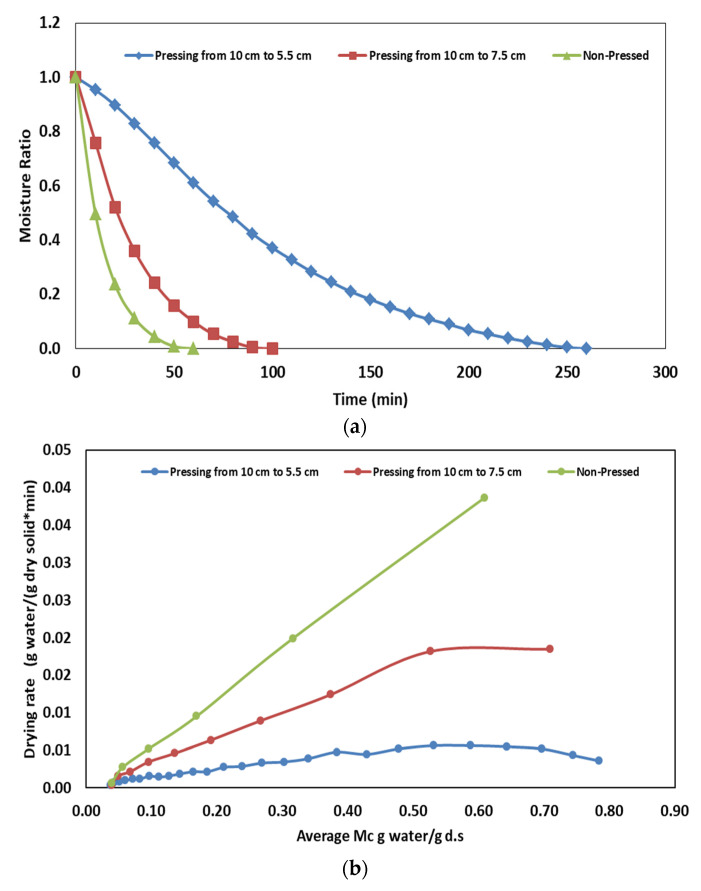
Effect of compaction density (**a**) drying behavior of olive pomace, (**b**) drying rate.

The effects of compaction density on the moisture curve and drying rate of olive pomace are given in Figure 3a and Figure 3b, respectively. Drying times for compaction densities of ~0.45, ~ 0.60 and ~0.82 g/cm^3^ were 60, 90 and 250 min, respectively. The highest drying rate was observed for the 10 cm thick sample (the unpressed sample, dc = 0.45 g/cm^3^) since air easily passes through the pores. However, as the amount of pressing increases, the air passage through the gaps in the bulk sample decreases and thus the drying rate slows down. The results revealed that the increase in the compaction density (from ~0.45 to ~0.82 g/cm^3^) increased the drying time approximately fourfold. Similarly, Elik [17] reported that the increase in the compaction density resulted in a longer drying time. 

### 3.2. Comparison of HA–RF, HA and RF Drying 

**Figure 4:**
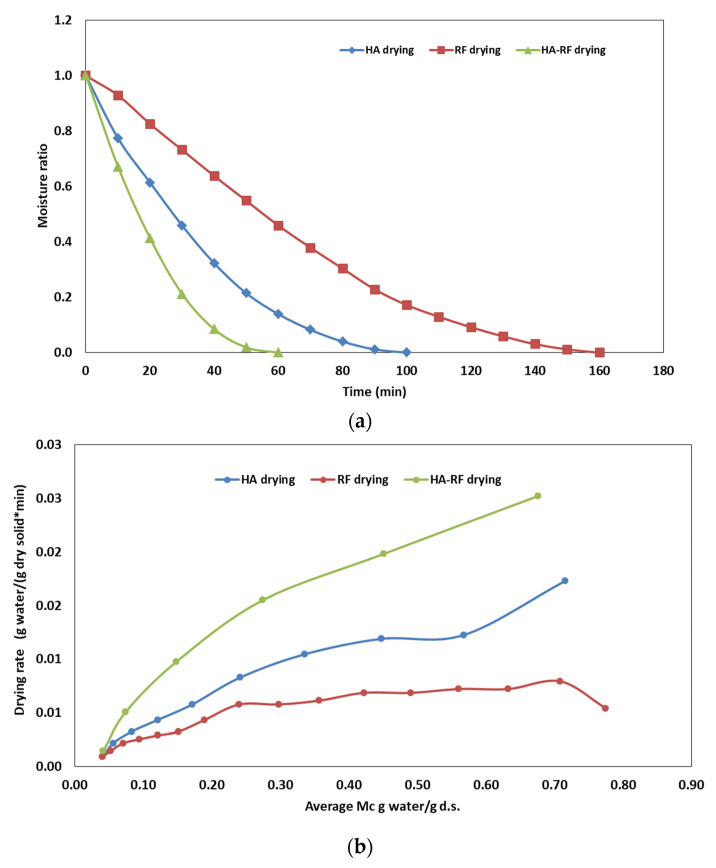
Comparison of HA, HA–RF and RF drying regarding the (**a**) drying behavior of olive pomace and (**b**) drying rate.

Figure 4a,b shows the effect of the drying methods (HA–RF, HA and RF drying) on the drying curve and the drying rate of olive pomace, respectively. The drying times for HA–RF, HA and RF drying methods were 60, 100 and 160 min, respectively. It was seen that the drying rate significantly increased following the RF application together with HA. Only RF drying had the longest drying time with a slow drying rate. This is basically because of a lack of driving force to carry out the water from the sample surface. Only HA drying, compared to only RF drying, has an increased drying rate. In this case, forced hot air has a better ability to remove water which is mostly loosely bound with a cellulosic composition of pomace. The combined effect of RF and HA resulted in a shorter drying time with an increased drying rate. It can be explained by a combination of the locational heating effect of RF and carrier effect of forced hot air. The results were consistent with the literature. Similarly, Elik [17] reported that the drying rate increased following HA–RF application in the drying of black carrot pomace. In another study, Zhang et al. [37] studied the HA–RF drying of in-shell walnuts and obtained that HA–RF drying was more effective compared to hot air drying. Also, Wang et al. [38] studied the HA–RF drying of macadamia nuts and obtained that HA–RF reduced the drying time by half compared to hot air drying.

**Figure 5:**
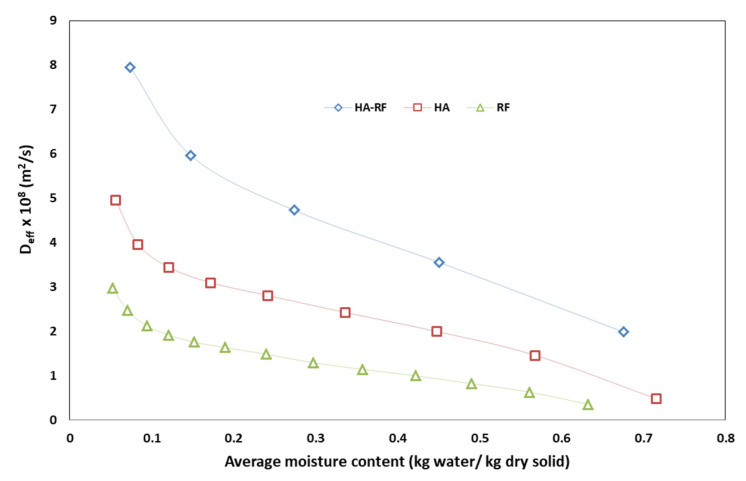
Variation in *D_eff_* with average moisture content using different drying methods.

Variations in the *D_eff_* values with average moisture contents for HA–RF, HA and RF drying methods are demonstrated in Figure 5. The results showed that the *D_eff_* values increased with a decrease in the average moisture content for all drying methods. The *D_eff_* values of olive pomace were found to be higher when the HA–RF drying system was used. This is probably due to the volumetric and fast heating ability of the RF heating and the presence of the convective air flow ensuring the swift removal of moisture from the surface. Therefore, moisture diffusion within olive pomace occurred faster in the HA–RF drying system. 

### 3.3. Physical and Chemical Properties of Crude Olive Pomace Oil

Some quality parameters of olive pomace oil obtained from the pomace dried using HA–RF are given in Table 1. Although there is no limit set by the European Commission regulation 2016/2095 [39] for the quality parameters of crude olive pomace oil, the characteristics given in Table 1 are very important for the determination of quality, cost of the refining process and the market price of the crude oil. The FFA content of the pomace oil (1.09 ± 0.02%) was lower than the contents reported by Yanık [6] for crude olive pomace oil extracted using both microwave (6.60%) and industrial extraction (5.80%) methods. Gomes and Caponio [15] also reported higher FFA contents (6.52–14.09%) for fourteen crude olive pomace oil samples compared to those obtained in this study. Kiralan et al. [40] expressed that the FFA content was 12.58% for a crude olive pomace oil obtained from a company in Turkey. However, Baysan, Koç, Güngör and Ertekin [14] reported much lower FFA contents (0.458 and 0.765%) for an olive pomace oil tray dried after pre-drying with a drum dryer compared to those previously reported in the literature. It can be concluded that the FFA content of the crude oil obtained from olive pomace dried via an HA–RF system was good in quality, because it is close to the limit (≤1%) set by the European Commission regulation 2016/2095 [39] for olive pomace oil. 

The PV (12.2 meq O_2_/kg oil) of the crude olive pomace oil was comparable with values reported in the literature, such as 10.6–38.8 meq O_2_/kg oil for industrial [15] and 10.20 meq O_2_/kg oil for laboratory-processed olive pomace [14]. Moreover, it was also lower than that of the specified level (<15 meq O_2_/kg oil) according to the European Commission regulation 2016/2095 [39] for olive pomace oil. The p-AnV of the crude olive pomace oil was 3.01 which was one of the lowest values reported for crude olive pomace oil in the literature. Gomes and Caponio [15] reported higher p-AnV values (from 8.74 to 14.12) for crude olive pomace oil than those reported in the current study. In another study, Gomes and Caponio [41] reported p-AnV in the range of 5.48–11.22. The totox value is calculated from the contribution of PV and p-AnV and shows the total oxidation status of the oils. The higher these values, the higher the oil’s oxidization. The totox value (27.4) of the crude olive pomace oil showed that drying the olive pomace with the HA–RF system did not cause a serious oxidative deterioration because the result was comparable with the values given in the literature [15]. 

The K_232_ value (1.43) of crude olive pomace oil was almost 2.5 times lower than the smallest value (3.88) given for crude pomace oil samples in a previous study by Gomes and Caponio [15]. The low K_232_ and K_270_ values were consistent with the PV and p-AnV of crude olive pomace oil. 

Polyaromatic hydrocarbons (PAH) are hazardous substances that can occur in foods through contamination and/or due to processing conditions, such as drying at high temperature [5]. Increasing PAH content in crude olive pomace oil is mainly due to incomplete combustion and direct exposure to combustion fumes during the traditional rotary drying process [6]. Benzo[a] pyrene is one of the heavy PAHs and it has been established to be a probable human carcinogen by the International Agency for Research on Cancer [42]. The European Commission regulation no. 208/2005 [43] has imposed a maximum benzo(a)pyrene limit of 2 µg/kg in oils and fats. Although it is a crude olive pomace oil, the benzo(a)pyrene value of the oil obtained from HA–RF dried pomace was significantly lower (<1 µg/kg oil) than the limit. Additionally, the benzo(a)pyrene value was lower than the benzo(a)pyrene content of crude olive pomace oil reported before: 1.67 µg/kg oil [6] and 13.55 µg/kg oil [4]. Hence, low initial PAH content is a critical issue in crude oils. In this respect, low benzo(a)pyrene contents in crude olive pomace show that HA–RF drying could be an alternative drying method to prevent PAH production during the drying stage of olive pomace. 

The intense green color of olive pomace oil is due to the presence of a high amount of chlorophyll pigment. The chlorophyll and total carotenoid content of crude olive pomace oil were obtained as 105.25 mg pheophytin a/kg oil and 2.85 mg/kg olive pomace oil, respectively. The chlorophyll content was higher than that reported earlier (77 mg pheophytin/kg crude olive pomace oil) by Serafini and Tonetto [44]. The chlorophyll content of crude olive pomace oil was higher but the carotenoid content was lower than the average values given for extra virgin olive oil (11.75 mg chlorophyll/kg oil, 8.21 mg carotenoid/kg oil) by Cho and Lee [45]. 

The color of the crude olive pomace oil is another physical characteristic directly influenced by the drying stage of olive pomace processing. The color parameters, L* (brightness), a* (redness) and b* (yellowness) values, of the oil extracted after HA–RF were 38.6, 7.5 and 62.56, respectively. The brightness of the crude oil obtained after HA–RF drying was better than that reported by Yanık [6] for both microwave (L*: 36) and conventionally (L*: 15.41) extracted crude olive pomace oil. It can be concluded that the HA–RF drying of olive pomace results in a good final crude oil quality in terms of color. 

The main fatty acids were oleic acid (65.14%) followed by linoleic (14.2%) and palmitic acid (13.4%) in crude olive pomace oil. The fatty acid composition of the crude olive pomace oil was consistent with the previously reported ones [6]. Hence, there was no adverse effect of HA–RF drying on the fatty acid composition of olive pomace oil.

## 4. Conclusions

Sample thickness, electrode distance and compaction density significantly affected the drying rate of olive pomace during hot-air-assisted radiofrequency drying. The application of RF together with hot air significantly reduced the drying time. The drying was accomplished within 60 min with this HA–RF system. The results of this study showed that HA–RF drying can be an alternative method for the rapid drying of olive pomace without adverse effects on the final oil quality. Moreover, the HA–RF drying of olive pomace was effective in reducing the process-induced PAH content and oxidation as well as improving the color quality. However, further studies on the energy efficiency of HA–RF drying and continuous HA–RF drying systems are recommended.

## Figures and Tables

**Table 1:** Physical and chemical properties of crude olive pomace oil.

Property	Crude Olive Pomace Oil
Free fatty acid content (% oleic acid)	1.09 ± 0.02
Peroxide value (meq O_2_/kg oil)	12.20 ± 0.07
*p*-AnV	3.01 ± 0.29
Totox	27.40 ± 0.14
PAH content (BaP, µg/kg oil)	<1 †
Chlorophyll content (mg pheophytin a/kg)	105.25 ± 0.84
Carotenoid content (mg/kg)	2.85 ± 0.02
UV absorption	
K232	1.43 ± 0.01
K270	0.23 ± 0.01
Color (CIE)	
L*	38.60 ± 0.03
a*	7.50 ± 0.01
b*	62.56 ± 0.21
Hue Angle	83.11 ± 0.01
Chroma	62.98 ± 0.12
Fatty acid composition (%) ‡	
C16:0	13.36 ± 0.19
C16:1	0.68 ± 0.02
C18:0	4.52 ± 0.03
C18:1	65.14 ± 0.54
C18:2	14.23 ± 0.07
C20:0	0.66 ± 0.03
C18:3	0.65 ± 0.01
C20:1	0.31 ± 0.01

† Below the detection limit of the instrument; ‡ Results expressed as percent over the total content (relative content).

## Data Availability

The data used to support the findings of this study can be made available by the corresponding author upon request.
